# An Adult Zebrafish Model Reveals that Mucormycosis Induces Apoptosis of Infected Macrophages

**DOI:** 10.1038/s41598-018-30754-6

**Published:** 2018-08-24

**Authors:** Azucena López-Muñoz, Francisco E. Nicolás, Diana García-Moreno, Ana B. Pérez-Oliva, María I. Navarro-Mendoza, Miguel A. Hernández-Oñate, Alfredo Herrera-Estrella, Santiago Torres-Martínez, Rosa M. Ruiz-Vázquez, Victoriano Garre, Victoriano Mulero

**Affiliations:** 1 Department of Cell Biology and Histology, University of Murcia, IMIB-Arrixaca, 30100 Murcia, Spain; 2 Department of Genetics and Microbiology, Faculty of Biology, University of Murcia, 30100 Murcia, Spain; 3 CONACYT - Centro de Investigación en Alimentación y Desarrollo, Hermosillo, Mexico; 4 Laboratorio Nacional de Genómica para la Biodiversidad, CINVESTAV-IPN, Irapuato, Mexico

## Abstract

Mucormycosis is a life-threatening fungal infection caused by various ubiquitous filamentous fungi of the Mucorales order, although *Rhizopus* spp. and *Mucor* spp. are the most prevalent causal agents. The limited therapeutic options available together with a rapid progression of the infection and a difficult early diagnosis produce high mortality. Here, we developed an adult zebrafish model of *Mucor circinelloides* infection which allowed us to confirm the link between sporangiospore size and virulence. Transcriptomic studies revealed a local, strong inflammatory response of the host elicited after sporangiospore germination and mycelial tissue invasion, while avirulent and UV-killed sporangiospores failed to induce inflammation and were rapidly cleared. Of the 857 genes modulated by the infection, those encoding cytokines, complement factors, peptidoglycan recognition proteins, and iron acquisition are particularly interesting. Furthermore, neutrophils and macrophages were similarly recruited independently of sporangiospore virulence and viability, which results in a robust depletion of both cell types in the hematopoietic compartment. Strikingly, our model also reveals for the first time the ability of mucormycosis to induce the apoptosis of recruited macrophages but not neutrophils. The induction of macrophage apoptosis, therefore, might represent a key virulence mechanism of these fungal pathogens, providing novel targets for therapeutic intervention in this lethal infection.

## Introduction

Mucormycosis is a life-threatening fungal infection caused by various ubiquitous filamentous fungi of the Mucorales order, although *Rhizopus* spp. and *Mucor* spp. are the most prevalent causal agents^1^. This fungal infection is a rare disease, but the advances in medical care and an increasingly aging population have resulted in an increased incidence^1,2^, which is also favored by the absence of effective pharmacological treatments compared to more frequent fungal infections, such as candidiasis and aspergillosis. The limited therapeutic options available together with a rapid progression of the disease and a problematic early diagnosis produce unacceptable mortality, higher than 90% in disseminated infections^3–5^. Mucormycosis is an infection affecting all ages, from premature neonates to older adults, with varying clinical forms^2^. Unlike other filamentous fungal pathogens that infect immunocompromised patients, Mucorales show a broader and more heterogeneous target population, which include immunocompetent persons. The most important underlying conditions that increase the risk of mucormycosis are diabetes mellitus, suppressed immunity (e.g., because of transplantation, malignancies, HIV, steroids, or neutropenia)^4,6^, elevated serum levels of iron under treatment with deferoxamine and the breakdown of the skin barrier by burns or local trauma injuries. Other risk factors for mucormycosis include intravenous drug abuse, malnutrition, premature birth, and long-term application of the broad-spectrum antifungal voriconazole^6^. This heterogeneity of risk factors and the reduced treatments available suggest that this currently rare infection hides the potential to become a devastating infection if a strain acquired a higher virulence. In fact, mucormycosis outbreaks have been increasingly frequent in recent years mainly due to traumas as in the tornado in Joplin(Missouri, USA), where 13 persons were affected^7^. Therefore, more studies to deepen in the biology of Mucorales and the progression of the Mucormycosis are required to identify virulence factors that could be targets of new drugs because most drugs in the market have no or low effectiveness against Mucorales.

Characterization of the infection by Mucorales requires a fungal model amenable for both genetic manipulation and large-scale genomic screening to identify pathogenicity factors, in addition to a suitable animal model that allows studying the disease progression and defense response of the animal to the fungus. Within Mucorales, *Mucor circinelloides* represent the best model for genetic manipulation because several genetic tools have been developed, including gene knockout by homologous recombination and gene knockdown by RNAi^8^. Moreover, it is the second most important agent in Mucormycosis and virulence has been linked to genetic differences between subspecies. A phylogenetic analysis based on multi-locus sequence typing established three different *M. circinelloides* subspecies corresponding to the *M. circinelloides f. lusitanicus* (*Mcl*), *M. circinelloides f. griseocyanus* (*Mcg*), and *M. circinelloides f. circinelloides* (*Mcc*). Strains of *Mcc* were found to be more prevalent among clinical *M. circinelloides* isolates, and more virulent than *Mcl* and *Mcg* strains in a diabetic murine model^9^. However, most common laboratories strains belong to *Mcl*, which shows a sporangiospore size dimorphism that is linked to virulence in larvae of the wax moth, *Galleria mellonella*. Thus, *Mcl* strains that produce larger asexual sporangiospores are more virulent in the wax moth host than strains that produce small sporangiospores. The existence of strains with different virulence offers an opportunity to identify the genes behind the ability to infect humans.

Several models have been developed to study mucormycosis in invertebrates and vertebrates, both *in vitro* and *in vivo*^9–14^. Mammalian models would represent the best model due to its proximity to humans, but large-scale studies are not possible because of ethical considerations, logistic restraints associated with their use and difficulties in studying the dynamics of host-pathogen interaction *in vivo*. In recent years, the zebrafish (*Danio rerio*) has become an accepted model because its immune system is similar to the human^15^. A zebrafish larval infection model has been recently established to study early innate immune response to *M. circinelloides* in a whole-animal system and in real-time *in vivo*^12^. Despite that this model has been used with an *Mcl* strain, which is avirulent in a diabetic murine model and shows reduced virulence in wax moth host, it mimics a range of aspects of human mucormycosis showing that macrophages and neutrophils were readily recruited to the site of infection. Although sporangiospore phagocytosis was observed, they hardly activated gene expression of pro-inflammatory cytokines^12^. This work extends the zebrafish model for mucormycosis to adult fish because of the difficulties to use strains that produce large sporangiospores in larvae, since they get frequently stuck in the narrow needles used for infection, and to have the possibility of analyzing both innate and adaptive immune responses. Our results confirm the link between sporangiospore size and virulence previously in wax moth^9^, larval zebrafish^12^ and murine^14^ models, but reveal a robust inflammatory response elicited after sporangiospore germination and mycelial tissue invasion. This host inflammatory response is characterized by neutrophil and macrophages mobilization from the kidney, the main adult fish hematopoietic organ, to the infection site, and the modulation of 857 genes related to immune response and iron metabolism, among others. Importantly, the results also uncover a previously unappreciated ability of mucormycosis to induce the apoptosis of recruited macrophages, what might represent a key virulence mechanism of this fungal infection providing a novel target for therapeutic intervention.

## Methods

### Ethics statement

The experiments complied with the Guidelines of the European Union Council (Directive 2010/63/EU) and the Spanish RD 53/2013. Experiments and procedures were performed as approved by the Consejería de Agua, Agricultura, Ganadería y Pesca de la CARM (authorization number #A13170801).

### Zebrafish husbandry

Zebrafish (*Danio rerio* H.) were obtained from the Zebrafish International Resource Center and mated, staged, raised and processed as described^16^.

### Mucor circinelloides strains and growth conditions

The leucine auxotroph R7B, derived from the (−) mating type *M*. *circinelloides* f. *lusitanicus* CBS 277.49, was used as the wild-type strain. The *M*. *circinelloides* f. *lusitanicus* strain of the (+) mating type NRRL3631 was used in virulence assays as an avirulent control. Both strains used in this study R7B and NRRL3631 (NRRL for simplicity) were grown at 26 °C in complete medium YPG, minimal YNB medium or semi-complex MMC medium^17^. Media were supplemented with L-leucine (20 μg/ml) or uridine (200 μg/ml), when required. The pH was adjusted to 4.5 for mycelial growth. Sporangiospores were inactivated by ultraviolet irradiation during 30 min at 254 nm (UV crosslinker, Hoefer).

### Zebrafish infection assays

Spores were collected from mycelium grown in YPG during five days, washed in distilled water and filtered using a cell strainer of 70 μm nylon mesh to eliminate rests of mycelium. These spores were kept in distilled water and used in the virulence assays during the next five days. Spore viability was assessed by plating on YPG medium, and no differences in viability were observed between R7B and NRRL. Zebrafish were intraperitoneally (i.p.) injected with live or UV-inactivated R7B (2.5 × 10^5^) or NRRL (2.5 × 10^5^ or 10^7^) sporangiospores/fish in groups of twenty specimens at 26 °C in 10-liter tanks. After the challenge, the fish were monitored every 12 h over a 7-day period for clinical signs of disease and mortality. At 16 hours post-infection (hpi), samples of the kidney and the abdominal organs were collected and processed for analysis of gene expression, while complete specimens were processed for histology (see below).

### Mouse macrophage cultures and treatments

The mouse macrophage cell line J774 was obtained from the European Collection of Authenticated Cell Cultures (ECACC) and grown in DMEM-high glucose supplemented with 10% heat-inactivated FCS, 100 U/mL penicillin and 100 µg/mL streptomycin at 37 °C in 5% CO_2_. Cells were incubated with 1:1 live or UV-killed sporangiospores from R7B and NRRL strains alone or in combination with 10 ng/ml lipopolysaccharide (LPS) from *Escherichia coli* 0111.B4 (Sigma-Aldrich) for 3 and 16 h, then washed twice with PBS and processed for RT-qPCR analysis as indicated below.

### Histology

Zebrafish were fixed in AZF solution (3% zinc chloride, 5.6% formol, 1% acetic acid) for four days at 4 °C, and descalcificated in EDTA (0.25 M, pH 8) for three days at 4 °C. Afterward, they were dehydrated, embedded in Paraplast Plus and sectioned at 5 µm. Serial sections were stained with periodic acid-Schiff stain (PAS) and specific antibodies to zebrafish myeloperoxidase (Mpx, #GTX128379, GeneTex), to zebrafish lymphocyte cytosolic protein 1 (Lcp1, #GTX124420, GeneTex) and to a fully conserved epitope of active caspase-3 (#AF835, R&D Systems) by using standard procedures^18,19^.

The quantification of Lcp1^+^, Mpx^+^, and Casp3^+^ cells was calculated as the mean ± SEM of the immunostained area/total area of 4 randomly distributed optical regions from 4 fish at ×200 magnification. The stained areas were measured by image analysis using a Nikon Eclipse E600 light microscope, an Olympus SC30 digital camera (Olympus Soft Imaging Solutions), and Leica Qwin software (Leica Microsystems).

### Library preparation and sequencing

Total RNA was extracted from the abdominal organs using TRIzol^®^ Plus RNA Purification System (Thermo Fisher Scientific) following the manufacturer’s instructions. To maximize target coverage, equal amounts of total RNA from three biological replicates of each strain were pooled for RNA-seq library construction. Both library preparation and sequencing was performed at Baseclear (Leiden, The Netherlands). The cDNA library was sequenced using Illumina HiSeq 2500 sequencer with one lane, 50 cycles, single-read sequencing strategy.

### RNA-seq data analysis

Quality control of the reads was carried out using FastQC v0.11.2^20^. Reads were trimmed to remove adapter sequences; the first 14 bases were removed from each read. Trimmed reads were mapped to the Ensembl (Flicek *et al*., 2014) Zebrafish genome release 75, using Bowtie2 version 2.1.0^21^ with “very-sensitive” and “end-to-end” parameters, then short reads with more than one hit were eliminated. The number of reads per gene generated was determined with HTSeq^22^, using the default “union” counting option. In order to identify the low expression genes, the number of reads per gene was normalized to counts per million (CPM), a simple measure of read abundance independent of the mean expressed transcript length and thus more comparable across different samples^23^. Only those genes with at least 2 CPM in at least one sample were considered as transcriptionally active and used for subsequent analyses. Differential expression of genes in fish inoculated with R7B spores (RDRZ) versus controls inoculated with PBS (PBS2) was estimated with the package edgeR (R version: 3.0.1, edgeR version: 3.2.4,^24^); using statistical methods based on pairwise comparison with common dispersion used to estimate variance between samples. For this analysis without biological replicates, the common dispersion was estimated of a conservative way from a list of putative housekeeping genes (determined for these samples, with a maximum fold change below 1.99 and transcriptionally expressed) and using the script: estimateDisp(dge,robust = TRUE,winsor.tail = c(0.05,0.2). The common dispersion calculated was 0.0840. Genes that exhibited an FDR ≤ 0.01 and a log2FoldChange > 1 (log2FC) were considered differentially expressed (DEG). A combination of David annotation system (http://david.abcc.ncifcrf.gov/), gene ontology (GO) database (www.geneontology.org) and manual curation were used to obtain the functional classification of the DEG. Enrichment analyses were performed using Blast2GO^25^, with an FDR ≤ 0.05. The enrichment ratio for each GO term was obtained by dividing the percentage of sequences of the test set versus the percentage of sequences of the reference set, where the reference set was the predicted genes in the genome, and the test sets were the up-regulated and down-regulated genes. The function to most specific terms was used to reduce the result-set of over-represented GO terms. The list of genes encoding proteins with transcription factor functions was downloaded from AnimalTFDB 2.0^26^. The gplots R library was used to generate the gene expression heatmaps (http://cran.r-project.org/web/packages/gplots/index.html).

### RT-qPCR

Total RNA extracted from zebrafish kidney and abdominal organs and mouse J774 macrophages as indicated above was treated with DNase I, Amplification grade (1 unit/μg RNA, Invitrogen) and the SuperScript III RNase H^−^ ReverseTranscriptase (Thermo Fisher Scientific) was then used to synthesize the first strand of cDNA with an oligo-dT_18_ primer from 1 μg of total RNA at 50 °C for 50 min. Real-time PCR was performed with an ABI PRISM 7500 instrument (Applied Biosystems) using SYBR Green PCR Core Reagents (Applied Biosystems). Reaction mixtures were incubated for 10 min at 95 °C, followed by 40 cycles of 15 s at 95 °C, 1 min at 60 °C, and finally, 15 s at 95 °C, 1 min at 60 °C and 15 s at 95 °C. For each mRNA, the gene expression was normalized to the ribosomal proteins S11 content in each sample using the Pfaffl method (30). Table 1 shows the primers used. In all cases, each PCR was performed with triplicate samples and repeated with at least three independent samples.

**Table 1:** Primers used in this study. The gene symbols followed the Zebrafish Nomenclature Guidelines (http://zfin.org/zf_info/nomen.html).

Gene	Accession number	Name	Nucleotide sequence (5′→3′)	Use
**Zebrafish primers**				
*il1b*	NM_212844	F5	GGCTGTGTGTTTGGGAATCT	gene expression
		R5	**TGATAAACCAACCGGGACA**	
*mpeg1*	NM_212737	F1	TGCGGCACAATCGCAGTCCA	gene expression
		R1	**ACAGCAAAACACCCATCTGGCGA**	
*il22*	NM_001020792	F	TACGCCAAAGGTGAAAAGACTA	gene expression
		R	AGATGAGTGGATTCGGGTATCT	
*tnfa*	NM_212859	F2	GCGCTTTTCTGAATCCTACG	gene expression
		R2	TGCCCAGTCTGTCTCCTTCT	
*ptgs2a*	NM_153657	F1	TGGATCTTTCCTGGTGAAGG	gene expression
		R1	GAAGCTCAGGGGTAGTGCAG	
*ptgs2b*	NM_001025504	F2	**CCCCAGAGTACTGGAAACCA**	gene expression
		R2	**ACATGGCCCGTTGACATTAT**	
*mpx*	NM_212779	F1	AGGCTCAGCAACACCTCCTA	gene expression
		R1	AGGGCGTGACCATGCTATAC	
**Mouse primers**				
*Il1b*	M54933	F	AGGGCGTGACCATGCTATAC	gene expression
		R	TACCAGTTGGGGAACTCTGC	
*Actb*	X_89920	F	GGCACCACACCTTCTACAATG	gene expression
		R	GTGGTGGTGAAGCTGTAGCC	

### Statistical analysis

Error bars indicate standard error of the mean (SEM). For gene expression experiments data are shown as mean ± SEM of three separate experiments. The differences between experimental treatments and control (PBS) were analyzed by Student’s *t* test. Log-rank (Mantel–Cox) test was used for the survival curves.

## Results

### An adult zebrafish model recapitulates the linkage between sporangiospore size dimorphism and virulence of *M. circinelloides*

**Figure 1:**
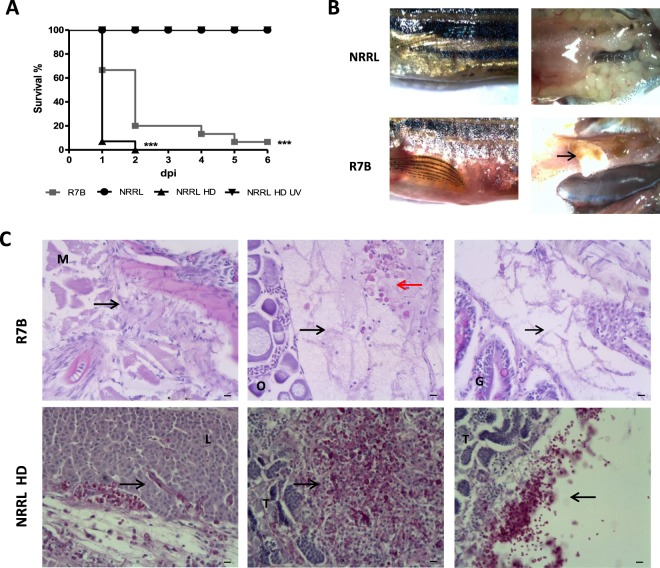
*M. circinelloides* R7B is highly pathogenic for zebrafish. Zebrafish were i.p. injected with 2.5 × 10^5^ or 10^7^ (high dose: HD) live or UV-killed sporangiospores of the R7B or NRRL strains. (**A**) The survival rates of infected zebrafish are shown. Each experimental group contained 20 zebrafish, and each experiment was performed in duplicate. ^*^P < 0.05; ^**^P < 0.01; ^***^P < 0.001. (**B**) Gross morphological examinations of adult zebrafish infected with *M. circinelloides* R7B and NRRL were imaged at 16 hpi. (**C**) Histological analysis of *M circinelloides-*infected zebrafish. Transverse sections were obtained from zebrafish injected with 2.5 × 10^5^ R7B spores/fish or 10^7^ NRRL spores/fish at 16 hpi. Note that the mycelium (black arrows) can invade several organs and promotes a strong leukocyte infiltration (red arrows). L, liver; G, gut; M, muscle; O, ovary; T, testis. Scale bars 10 µm

Adult fish were i.p. infected with different doses of sporangiospores from the R7B and NRRL strains, which produce large and small sporangiospores, respectively. The results showed that 2.5 × 10^5^ sporangiospores consistently resulted in 100% mortality of zebrafish infected with R7B as early as 24 hpi and no mortality with the same dose of NRRL sporangiospores (Fig. 1A). This clear difference in virulence between strains that produce big and small sporangiospores was previously observed in infections of *Galleria mellonela* larvae^9^. However, a much higher dose of NRRL sporangiospores (10^7^) was able to kill zebrafish as effective as 2.5 × 10^5^ sporangiospores of R7B, the effect being dependent on sporangiospores viability (Fig. 1A). Moribund fish showed redness of the abdomen and petechial hemorrhaging, and the mycelium was observed invading different abdominal organs (Fig. 1B). Histological examination confirmed germination of R7B sporangiospores and hyphal invasion, and frequently vacuolization and disorganization, of various organs, including muscle, liver, intestine, ovarium, and testis (Fig. 1B,C). Although similar results were found when using the high dose of sporangiospores of the NRRL strain, abundant sporangiospores remained inside the peritoneal cavity and did not germinate (Fig. 1C).

### Zebrafish transcriptome analysis in response to *M. circinelloides* infection reveals genes with putative functions in mucormycosis

To determine zebrafish transcriptomic response to infection with *M. circinelloides* R7B strain, we prepared and sequenced two RNA libraries from zebrafish inoculated with a 2.5 × 10^5^ sporangiospores of the R7B strain (RDRZ) or buffer (PBS2). We obtained about of 15 and 14 million short reads of a length of 50 nt and an average quality score (Phred) of 39.19 and 39.22 for PBS2 and RDRZ libraries, respectively (Table S1). After trimming, the total of reads with a length mean of 36 nt for each sample were mapped to the zebrafish reference genome. After eliminating ambiguous reads (with more than one hit to the genome), we obtained 9,372,491 reads (64.5%) and 9,644,092 reads (62%) for RDRZ and PBS2 libraries, respectively (Table S1). Overall transcriptional activity, using normalized reads with at least 2 CPM in at least one sample, indicated that 16,071 genes are transcriptionally active, representing about 60.7% of predicted genes in the *D. rerio* genome.

**Figure 2:**
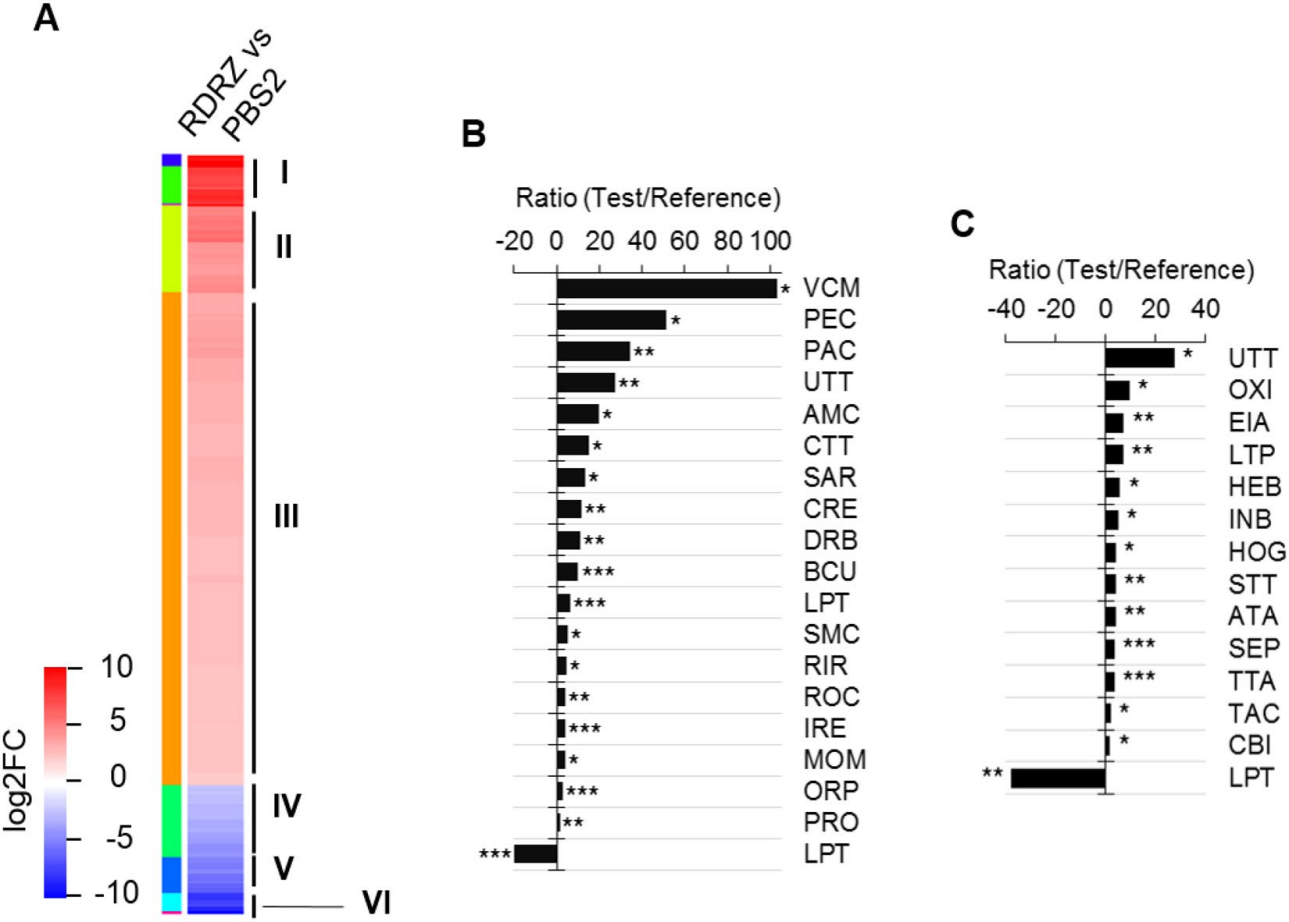
Differential expression analysis. (**A**) Heat-map shows the 857 differentially expressed genes clustered by their expression levels in response to *M. circinelloides* R7B strain infection. Roman numbers and left column colors indicate independent clusters. Color key indicates the log2 fold change values. (**B**) The graph shows the most specific biological process enriched. (**C**) The graph shows the most specific molecular function enriched. In (**B**,**C**), X-axis indicates the enrichment ratio obtained by dividing the sequences percentage of the test set versus the sequences percentage of the reference set (see methods section). Positive and negative values, respectively represent biological processes and molecular function enriched in up-regulated, and down-regulated genes. AMC, aminoglycan catabolic process; ATA, ATPase activity; BCU, blood coagulation; CBI, calcium ion binding; CRE, cellular response to estrogen stimulus; CTT, carbohydrate transmembrane transport; DRB, defense response to bacterium; EIA, endopeptidase inhibitor activity; HEB, heme binding; HOG, hydrolase activity, hydrolyzing O-glycosyl compounds; INB, iron ion binding; IRE, immune response; LPT, lipid transport; MOM, monocarboxylic acid metabolic process; ORP, oxidation-reduction process; OXI, oxidoreductase activity; PAC, protein activation cascade; PEC, peptidoglycan catabolic process; PRO, proteolysis; RIR, regulation of immune system process; ROC, response to organic cyclic compound; SAR, sarcomere organization; SEP, serine-type endopeptidase activity; SMC, small molecule catabolic process; STT, sodium ion transmembrane transporter activity; TAC, transporter activity; TTA, active transmembrane transporter activity; UTT, urea transmembrane transport; VCM, ventricular cardiac myofibril assembly. Asterisks indicate the statistical significance level, FDR ≤ 0.001 (***), FDR ≤ 0.01 (**) and FDR ≤ 0.05 (*).

To study the zebrafish transcriptional response to infection with *M. circinelloides* R7B strain, we compared the RDRZ sample versus the control (PBS2). A total of 857 differentially expressed genes were detected, from which 712 were up-regulated and 145 down-regulated (Fig. 2A and Additional file 1). A combination of David annotation system and GO database allowed to associate a functional annotation and/or molecular function to 83.9% of differentially expressed genes (Additional file 1 and Fig. S1).

To identify the biological process and molecular functions involved in the Zebrafish-*M. circinelloides* interaction, we performed a GO enrichment analysis using Blast2GO. The GO terms with an FDR ≤ 0.05 were considered enriched. Our analysis showed enrichment of 52 biological processes and 29 molecular functions in the up-regulated genes and only two biological processes and three molecular functions in the down-regulated genes (Additional file 2). Subsequently, the function of most specific terms was used to reduce the over-represented GO terms. This analysis indicated that the up-regulated genes were enriched in biological processes and molecular functions related to peptidoglycan catabolic, protein activation cascade, defense response to bacterium, blood coagulation, immune response, regulation of immune response, urea transport activity, oxidoreductase activity, endopeptidase inhibitor, lipid transporter activity, serine-type endopeptidase activity, transmembrane transporter activity, and calcium ion binding among others. In contrast, the down-regulated genes were enriched only in biological process and molecular functions related to lipid transport activity (Fig. 2B,C and Additional file 2).

The clustering by expression levels of the 857 genes grouped them in 6 clusters (Fig. 2A). Most of the transcriptional activity in response to infection by the R7B strain was displayed on the up-regulated genes (clusters I, II, III). Cluster I comprised 57 genes strongly induced related to iron homeostasis, cytoskeleton and muscle contraction. Like cluster I, cluster II included 99 induced genes related mainly to cytoskeleton and muscle contraction. Interestingly, the biggest cluster III was constituted by 556 slightly induced genes involved in protein activation cascade, regulation of immune response and transport. On the other hand, the down-regulated genes were grouped in the clusters IV, V and VI. Custer IV included 80 slightly repressed genes related to oxidoreductase activity. Cluster V comprised 41 repressed genes involved mainly in lipid transport. Cluster VI formed by 24 strongly repressed, however, their functions were not clear (Fig. 2A and Additional file 1). In addition, an enrichment analysis of each cluster showed GO terms enriched only for the clusters III and V. Cluster III the most enriched categories were related to protein activation cascade, coagulation, response to estrogen, regulation of immune response, response to wounding, transport activity, oxidoreductase activity, endopeptidase inhibitor activity, enzyme inhibitor activity and lipid transport activity among others. Also, cluster V was enriched in genes encoding proteins involved in lipid transport, response to estradiol and single-organism transport (Additional file 1).

Of the 857 differentially expressed genes, 26 were enriched in GO terms related to immune response and/or regulation of immune system response. As shown in Table 2 this group included up-regulated genes related to chemokine signal transduction, complement system pathway, major histocompatibility complex, cytokines system, proteoglycan metabolism, pathogen recognition, activation of innate immunity and Lck/Yes novel (LYN) tyrosine kinase. Interestingly, our enrichment analysis showed a genes group enriched for a GO term related to defense response to bacterium. This group comprised six genes encoding proteins with functions connected to interleukin system, peptidoglycan recognition, ribonuclease, pathogen recognition and activation of innate immunity through toll-like receptor 5b, antimicrobial peptide, and iron homeostasis. Another of the biological processes enriched in the infection by *M. circinelloides* was the protein activation cascade process, which encompassed four induced genes mainly related to the complement system including the complement C2, complement factor B and the mannan-binding lectin serine peptidase 2 genes (Table 2).

**Table 2:** Defense-related genes group.

Gene Id	Gene description	GO term enriched^a^	log2FC
ENSDARG00000087474	C-C chemokine receptor type 6	A	4.26
ENSDARG00000055441	similar to MHC class II alpha chain	A	4.14
ENSDARG00000074487	growth-regulated alpha protein-like	A	3.93
ENSDARG00000039516	complement component 8, alpha polypeptide	A	3.62
ENSDARG00000058389	chemokine CCL-C5a	A	3.62
ENSDARG00000016319	complement component 9	A	3.52
ENSDARG00000039351	chemokine (C-C motif) ligand 19b precursor	A	3.49
ENSDARG00000054202	hexose-binding lectin 4	A	3.32
ENSDARG00000075045	chemokine CXCL-C1c	A	2.95
ENSDARG00000039517	complement component 8, beta polypeptide	A	2.88
ENSDARG00000089534	C-C motif chemokine 21-like	A	2.87
ENSDARG00000025311	deleted in malignant brain tumors 1 protein-like	A	2.42
ENSDARG00000028163	proteoglycan 4	A	2.39
ENSDARG00000089050	deleted in malignant brain tumors 1 protein	A	2.34
ENSDARG00000036588	major histocompatibility complex class I ZE like precursor	A, B	2.93
ENSDARG00000056200	similar to vertebrate ATP-binding cassette, sub-family B (MDR/TAP), member 9 (ABCB9)	A, B	2.67
ENSDARG00000052322	toll-like receptor 5b	A, B, C	3.03
ENSDARG00000019772	complement C2	A, B, D	3.70
ENSDARG00000055278	complement factor B	A, B, D	3.05
ENSDARG00000007988	mannan-binding lectin serine peptidase 2	A, B, D	2.64
ENSDARG00000005419	interleukin 1, beta	A, C	3.56
ENSDARG00000070669	si:dkey-269d20.3; 7tm-1, G protein-coupled receptor domain containing protein	B	4.96
ENSDARG00000031715	tyrosine-protein kinase Lyn	B	2.36
ENSDARG00000053227	preprohepcidin 2; hepcidin antimicrobial peptide 1	C	6.83
ENSDARG00000015626	peptidoglycan recognition protein 6	C	3.94
ENSDARG00000043196	ribonuclease like 2	C	2.62
ENSDARG00000062998	peptidoglycan recognition protein 2	C	2.60
ENSDARG00000042684	serine (or cysteine) proteinase inhibitor, clade C (antithrombin), member 1	D	2.44

^a^GO terms enriched using Blast2GO with an FDR ≤ 0.05. **A**, Immune response; **B**, regulation of immune system process; **C**, defense response to bacterium; **D**, protein activation cascade.

### Sporangiospore germination and host infection induce a strong inflammatory response

**Figure 3:**
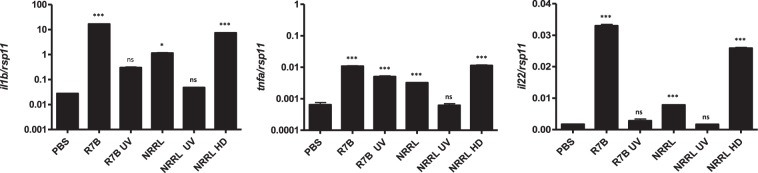
R7B is more potent than NRRL inducing immune-related gene expression in zebrafish. Zebrafish were i.p injected with 2.5 × 10^5^ or 10^7^ (high dose: HD) live or UV-inactivated sporangiospores of the R7B or NRRL strains. At 16 hpi, the expression of the indicated immune-related genes was analyzed by RT-qPCR in the infection site and compared to those of control fish injected with PBS (n = 4). ^*^P < 0.05; ^***^P < 0.001. ns, nonsignificant.

**Figure 4:**
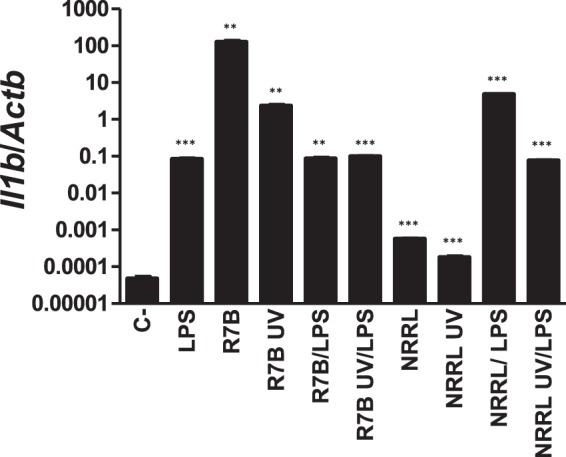
*M circinelloides* can activate mouse macrophages. IL-1β mRNA levels assayed by RT-qPCR in the macrophage cell line J774 at 3 hpi with live or UV-killed R7B and NRRL strains (MOI = 1) alone or in combination with 10 ng/ml LPS. ^**^p < 0.01, ^***^P < 0.001. ns, nonsignificant.

RT-qPCR analyses confirmed the local strong immune response to the infection and validated the transcriptomic analysis (Fig. 3). Thus, the R7B strain drastically increased the transcript levels of interleukin-1β (*il1b*), tumor necrosis factor α (*tnfa*) and *il22* genes, which encodes major pro-inflammatory cytokines. Interestingly, sporangiospores germination and host infection was required to promote a local inflammatory response, since challenge with live NRRL and UV-killed R7B and NRRL sporangiospores failed to increase the mRNA levels of *il1b*, *tnfa*, and *il22*, while a higher dose of NRRL sporangiospores, which could kill the fish, promoted a similar effect than a lower dose of highly virulent R7B. These results were confirmed in mouse J774 macrophages, where live R7B sporangiospores induced higher *Il1b* transcript levels than NRRL independently of costimulation with LPS (Fig. 4).

### *M. circinelloides* promotes myeloid cell depletion in the kidney

**Figure 5:**
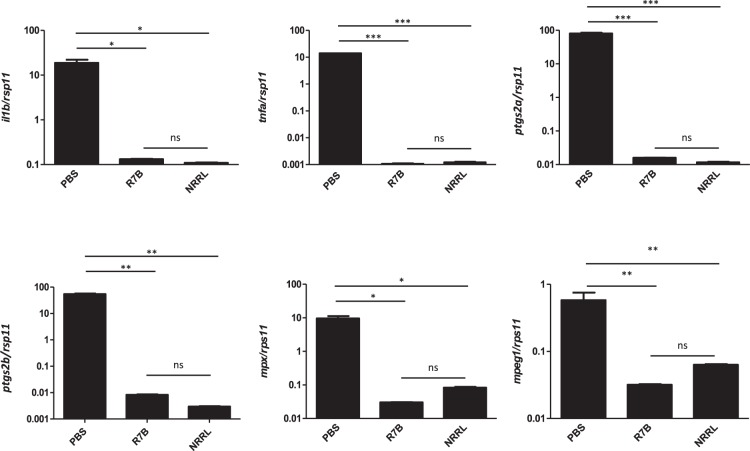
*M. circinelloides* inhibits the expression of immune-related genes in the head kidney of zebrafish. The mRNA levels of the genes coding for the indicated pro-inflammatory and neutrophil (*mpx*) and macrophage (*mpeg1*) markers were determined by RT-qPCR at 16 hpi in the head kidney from zebrafish challenged with 2.5 × 10^5^ R7B spores/fish or 10^7^ NRRL spores/fish (n = 8). ^*^P < 0.05; ^**^P < 0.01; ^***^P < 0.001. ns, nonsignificant.

**Figure 6:**
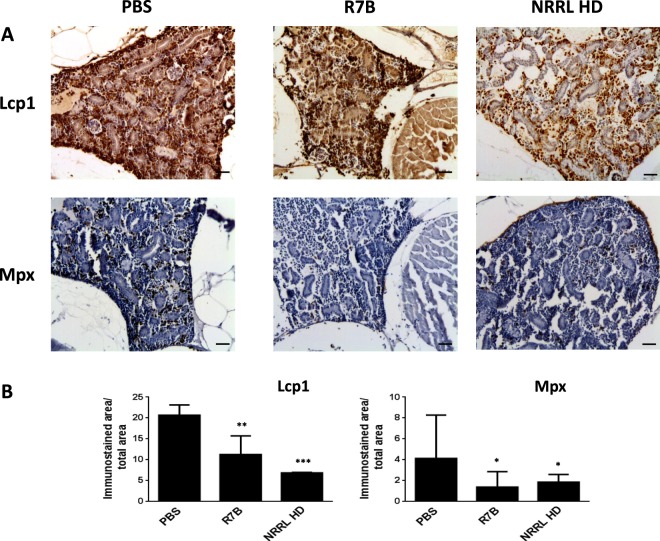
*M. circinelloides* promotes neutrophils and macrophages depletion in the head kidney. (**A**) Representative sections of zebrafish head kidneys infected with 2.5 × 10^5^ R7B spores/fish at 16 hpi and immunostained with anti-Lcp1 (pan-leukocyte marker) and anti-Mpx (neutrophil marker) antibodies. Note the robust depletion of myeloid cells. Scale bars 20 µm. (**B**) Quantification of Lcp1^+^ and Mpx^+^ cells is shown as the mean ± SEM of the immunostained area/total area of 4 randomly distributed optical areas from 4 fish at ×200 magnification. ^*^P<0.05; ^**^P<0.01; ^***^P<0.001.

The above results prompted us to examine the impact of infection at systemic levels. Therefore, we analyze the gene expression levels of genes encoding major pro-inflammatory mediators in the kidney, which is the principal hematopoietic organ in adult zebrafish. Infection with both strains resulted in dramatically reduced mRNA levels of *il1b*, *tnfa*, prostaglandin-endoperoxide synthase 2a (*ptgs2a*) and *ptgs2b* compared with fish injected with PBS (Fig. 5). Notably, the transcript levels of genes encoding neutrophil (myeloperoxidase, Mpx)^27^ and macrophage (macrophage expressed 1, Mpeg1)^28^ markers, also robustly declined (Fig. 5), suggesting the depletion of both cell types in the kidney. This was confirmed by staining neutrophils and macrophages with antibodies to zebrafish Lcp1 and Mpx that consistently revealed a drastic reduction of macrophages (Lcp1^+^/Mpx^−^) and neutrophils (Lcp1^+^/Mpx^+^) at 16 hpi in the kidney of R7B infected fish (Fig. 6A,B).

### *M. circinelloides* infection induces apoptosis of recruited macrophages in the infection site

**Figure 7:**
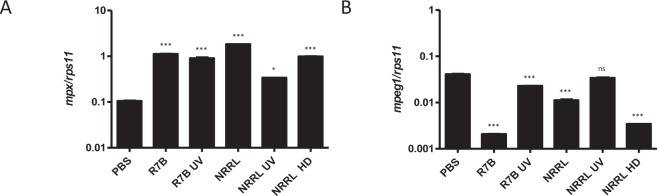
*M. circinelloides* altered the expression profile of gene encoding neutrophil and macrophages markers in the infection site. The mRNA levels of the genes coding for neutrophil (*mpx*) (**A**) and macrophages (*mpeg1*) (**B**) markers were determined by RT-qPCR at 16 hpi in the head kidney of zebrafish challenged with live or UV-killed 2.5×10^5^ R7B spores/fish and 2.5×10^5^ or 10^7^ (high dose, HD) NRRL spores/fish (n=4). ^*^P<0.05; ^***^P<0.001. ns, nonsignificant.

**Figure 8:**
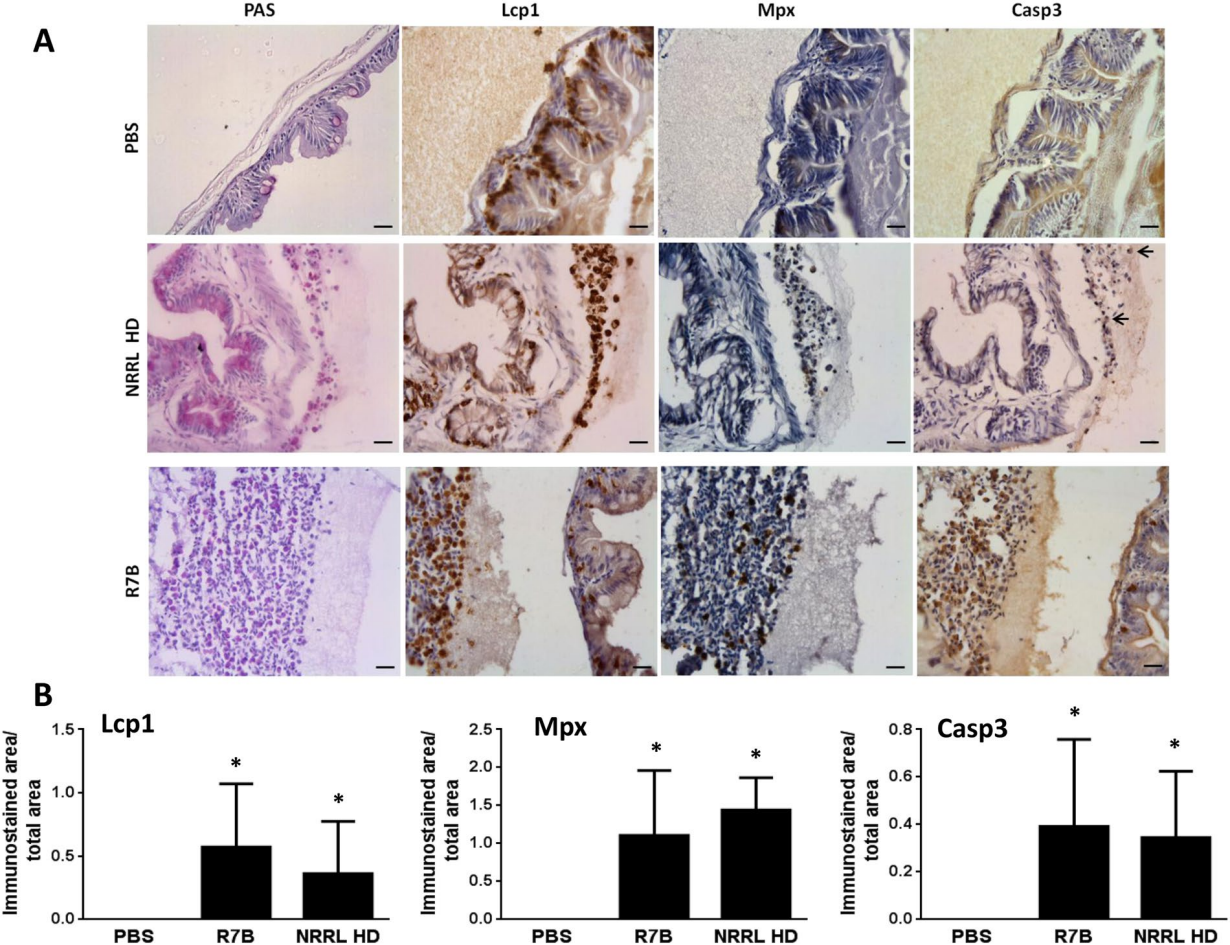
*M. circinelloides* promotes the recruitment and apoptosis of macrophages in the infection site. (**A**) Representative serial sections of zebrafish head kidneys challenged with PBS, 2.5×10^5^ R7B spores/fish and 10^7^ (high dose, HD) NRRL spores/fish at 16 hpi and immunostained with anti-Lcp1, anti-Mpx, and anti-active Casp3 antibodies. Infiltrated leukocytes (black arrows) at 16 hpi were PAS^+^, Lcp1^+^ and most of them Casp3^+^ in the fish infected with the virulent strain (R7B) and the HD of the avirulent NRRL strain. Scale bars 20 µm. (**B**) Quantification of Lcp1^+^, Mpx^+,^ and Casp3^+^ cells is shown as the mean ± SEM of the immunostained area/total area of 4 randomly distributed optical regions from 4 fish at ×200 magnification. ^*^P<0.05.

We then analyzed the expression of the genes encoding Mpx and Mpeg1 in the infection site. Strikingly, the transcript levels of *mpx* increased in infected fish with both live and UV-killed sporangiospores from both strains (Fig. 7A), whereas those of *mpeg1* robustly declined in fish infected with R7B and, to some extent, with the high dose of NRRL (Fig. 7B); the two conditions that resulted in high fish mortality. This result might suggest that infection promoted macrophage killing. Histological analysis using the anti-Lcp1 and anti-Mpx antibodies revealed that numerous macrophages (Lcp1^+^/Mpx^−^), but not neutrophils (Lcp1^+^/Mpx^+^), were apoptotic, i.e. Casp3^+^, in fish infected with the R7B strain and with the high dose of the NRRL strain (Fig. 8A,B), confirming the gene expression analysis.

## Discussion

Although mucormycosis has historically been considered a rare disease, at present is the third most prevalent fungal infection^29^. Although both *in vitro* and *in vivo* models in invertebrates and vertebrates have been established, additional models able to shed light into the early stage of infection, when the transition to filamentous growth occurs, will help to design appropriate prophylactic in targeted populations. We have developed here an adult zebrafish model of mucormycosis by i.p. injection of sporangiospores that recapitulates the link between sporangiospore size and virulence previously reported in wax moth^9^ and larval zebrafish^30^ models. This observation confirms the relevance of identifying therapeutic targets to inhibit fungal germination and potentiate protective immunity to sporangiospores before the transition to mycelial growth^31^.

Our model also revealed a robust inflammatory response at the infection site characterized by the upregulation of genes encoding major pro-inflammatory cytokines, such as Il1b, Tnfa, Il22, several chemokines and chemokine receptors, complement factors, and pattern recognition receptors (PRR). It is known that invasive filamentous growth induces IL1B and TNFA release by human neutrophils through a TLR2-signaling pathway^32^, while human monocyte-derived dendritic cells release IL1B, TNFA and IL23 via dectin-1 signaling^33^. However, hyphae are highly resistant to oxidative damage^32^. Our transcriptomic analysis revealed the induction of genes encoding complement factors, including complement factor B (Cfb) and mannan-binding lectin serine peptidase 2 (Masp2), which are involved in the activation of the alternative and lectin pathways, respectively. This observation, together with the ability of *M. circinelloides* sporangiospores to activate such pathways^34^, suggests a role of complement activation on the control of *M. circinelloides* germination and mycelial growth. However, functional studies will be required to demonstrate a role for complement in the control of mucormycosis. Furthermore, genes encoding two peptidoglycan recognition proteins (PGRP), were also induced in infected fish. Although these proteins are involved in bacterial clearance, a recent report showed that *Candida albicans* infection induces PGRP2 in human corneal epithelial cells though dectin-1/NF-κB signaling and, more importantly, that PGRP2 suppress colony-forming units of *C. albicans in vitro*^35^. Finally, several genes related to iron homeostasis, including the antimicrobial peptide hepcidin, were highly induced by infection, further confirming the critical role of iron acquisition by Mucorales in the fate of the infection^36^.

Our results also showed a robust neutrophil and macrophage mobilization from the kidney, the main adult fish hematopoietic organ, to the infection site. Curiously, while the induction of the potent inflammatory response required sporangiospores germination and mycelia colonization of host tissues, neutrophil mobilization was similarly elicited by both strains as well as by alive and UV-killed sporangiospores. Similarly, recruitment of macrophages and neutrophils to viable and UV-killed was also reported at real-time using a larval zebrafish model, although both phagocyte types showed clustering exclusively around viable spores, what may inhibit spore dissemination^30^. In contrast, spores from other fungi, such as *Rhizopus oryzae* and *Aspergillus fumigatus*, are unable to promote neutrophil chemotaxis *in vitro*^37^. Although these differences may be related to the particular fungal pathogens, the *ex vivo* systems are unable to model the critical interactions between macrophages and neutrophils that occur *in vivo*. For example, in a mouse model of pulmonary *A. fumigatus* infection, it has been shown that neutrophil recruitment depends on the release of IL-1α and CXCL1 by CCR2^+^ monocytes^38^, highlighting the need of *in vivo* fungal infection models.

The most important observation of our study is the previously unappreciated ability of mucormycosis to induce the apoptosis of recruited macrophages and the robust depletion of both macrophages and neutrophils from the hematopoietic compartment. Notably, the apoptosis of recruited macrophages was caused by the virulent R7B strain but also by a high dose of the less pathogenic NRRL, suggesting that germination is required for induction of macrophage apoptosis. Similarly, manipulation of neutrophils and macrophages apoptosis and myelopoiesis has been shown to be critical for the *in vivo* clearance of *A. fumigatus*^39^ and Pneumocystis^40^ lung infections, and *C. albicans* renal infection^41^. Also, *Cryptococcus neoformans* can induce macrophage apoptosis in a capsule-dependent manner, suggesting the presence of molecules in the capsule that trigger apoptosis^42^. Collectively, these results suggest that induction of macrophage apoptosis might represent a key virulence mechanism of mucormycosis and, therefore, provides a novel target for therapeutic intervention.

In summary, we have established an adult zebrafish mucormycosis model highly complementary to the mouse one which allows reconstituting the complex interactions between macrophages and neutrophils in response to fungal infections and, with its amenable genetic and pharmacological manipulation, will contribute to understanding the mechanisms involved in the manipulation of innate immunity by Mucorales. Using this model, we have confirmed the link between sporangiospore size and virulence, revealed a strong inflammatory response elicited after sporangiospore germination and mycelial tissue invasion, which is characterized by neutrophil and macrophages recruitment, and the modulation of 857 genes related to immune response and iron metabolism, and uncovered the ability of mucormycosis to induce the apoptosis of recruited macrophages.

## Electronic supplementary material

Figure S1

Additional File 1

Additional File 2

## Data Availability

All data generated or analyzed during this study are included in this published article and its Supplementary Information files. Illumina RNA sequencing data have been deposited in the NCBI Sequence Read Archive (SRA) under accession number SRP132190.
