# Supplementary material for: An Adult Zebrafish Model Reveals that Mucormycosis Induces Apoptosis of Infected Macrophages
Source: Sci Rep. 2018 Aug 24;8:12802. doi: 10.1038/s41598-018-30754-6 (PMC6109148; doi:10.1038/s41598-018-30754-6)
Supplement: Supplementary file 1 — Figure S1 [file 41598_2018_30754_MOESM1_ESM.pdf]

AN ADULT ZEBRAFISH MODEL REVEALS THAT MUCORMYCOSIS INDUCES APOPTOSIS OF INFECTED MACROPHAGES

Azucena López-Muñoz**,** Francisco E. Nicolás, Diana García-Moreno, Ana B. Pérez-Oliva, María I. Navarro-Mendoza, Miguel A. Hernández-Oñate, Alfredo Herrera-Estrella, Santiago Torres-Martínez, Rosa M. Ruiz-Vázquez, Victoriano Garre and Victoriano Mulero

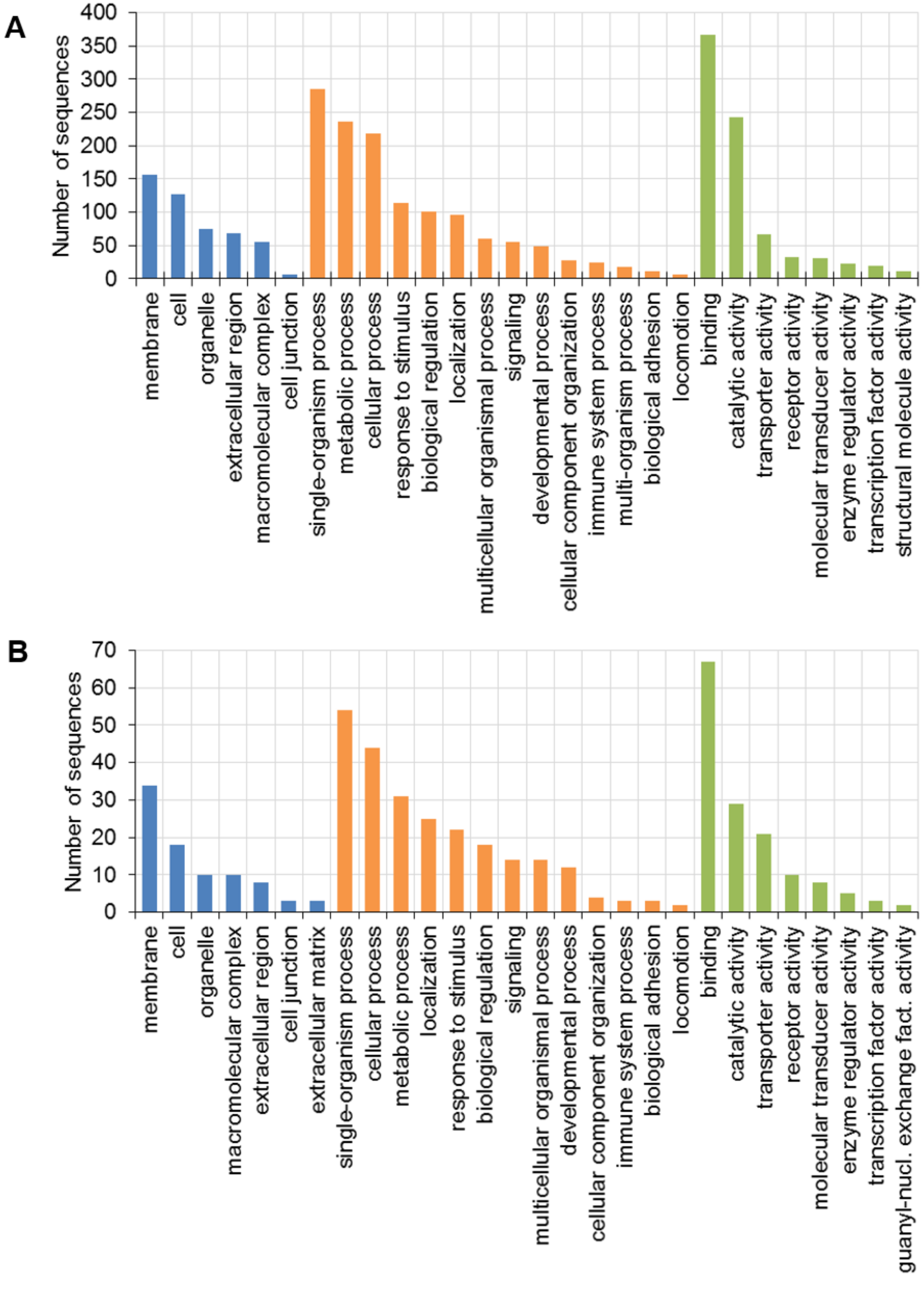

Figure S1. Gene Ontology (GO) annotation at level 2 of differentially expressed genes. **A**. Graph shows the mapping of the up-regulated genes on GO terms. **B**. Graph shows the mapping of the down-regulated genes on GO terms. The categories of cellular component, biological process and molecular function are indicated in blue, orange and green bars, respectively.
